# Prospecting for Microelement Function and Biosafety Assessment of Transgenic Cereal Plants

**DOI:** 10.3389/fpls.2018.00326

**Published:** 2018-03-15

**Authors:** Xiaofen Yu, Qingchen Luo, Kaixun Huang, Guangxiao Yang, Guangyuan He

**Affiliations:** ^1^The Genetic Engineering International Cooperation Base of Chinese Ministry of Science and Technology, Key Laboratory of Molecular Biophysics of Chinese Ministry of Education, College of Life Science and Technology, Huazhong University of Science & Technology, Wuhan, China; ^2^School of Chemistry and Chemical Engineering, Huazhong University of Science & Technology, Wuhan, China

**Keywords:** biosafety, cereal, mineral, transgene, trace element

## Abstract

Microelement contents and metabolism are vitally important for cereal plant growth and development as well as end-use properties. While minerals phytotoxicity harms plants, microelement deficiency also affects human health. Genetic engineering provides a promising way to solve these problems. As plants vary in abilities to uptake, transport, and accumulate minerals, and the key enzymes acting on that process is primarily presented in this review. Subsequently, microelement function and biosafety assessment of transgenic cereal plants have become a key issue to be addressed. Progress in genetic engineering of cereal plants has been made with the introduction of quality, high-yield, and resistant genes since the first transgenic rice, corn, and wheat were born in 1988, 1990, and 1992, respectively. As the biosafety issue of transgenic cereal plants has now risen to be a top concern, many studies on transgenic biosafety have been carried out. Transgenic cereal biosafety issues mainly include two subjects, environmental friendliness and end-use safety. Different levels of gene confirmation, genomics, proteomics, metabolomics and nutritiomics, absorption, metabolism, and function have been investigated. Also, the different levels of microelement contents have been measured in transgenic plants. Based on the motivation of the requested biosafety, systematic designs, and analysis of transgenic cereal are also presented in this review paper.

## Introduction

Rice, corn, and wheat are the three main staples, providing 60 percent of the world's food energy intake. The United States Department of Agriculture (USDA) estimates that the 2017/2018 global production will be 481.04 million metric tons of rice, 1031.86 million metric tons of corn, and 753.09 million metric tons of wheat. The United Nations Food & Agriculture Program has declared that global production of food, feed and fiber might have to be approximately doubled due to the growing global population, and this is greatly challenging traditional agriculture. Modern agriculture would benefit tremendously from the application of genetic engineering technology to enhance food crop quality, to increase yield production and also to improve resistance.

Transgenic cereal crops, known as genetically modified (GM) crops, have gained attention worldwide since they have emerged. By introducing bioengineering technology into crop breeding issues, GM crops with improved quality, enhanced resistance to biotic or abiotic stresses, increased yield, or reduced harmful components were generated (Toriyama et al., 1988; Gordon-Kamm et al., 1990; Vasil et al., 1992). Compared with the conventional breeding approach, the molecular breeding technique showed more efficiency at directly improving crop quality. However, exogenous gene transformation and expression may cause unforeseen modifications of the genome, and the commercially grown and promoted GM crops have to be subjected to safety assessments. Therefore, reliable safety evaluations are vital for the commercialization and public acceptance of GM cereal crops as well as their products (Tabashnik et al., 2013; Jin et al., 2015; Pulla, 2016). In 2016, the USA National Academies of Sciences, Engineering, and Medicine reported on genetically engineered crops including experiences and prospects, and the results showed that transgenic cereals are safe (Gloud et al., 2016). In the same year, the UK Royal Society also made an announcement that currently available GM food is safe to eat as non-GM food, and more than 100 of the world's top scientists have taken a firm position in the controversy over genetically modified organisms (GMO) by publishing an open letter to the international Greenpeace organization as well as those who campaign against GM crop marketing. So far, several international authoritative organizations, including the Organization for Economic Cooperation and Development (OECD) and the World Health Organization (WHO), have set standards for safety assessments including GM crops and products (Kitta, 2013). The substantial equivalent principle, which was first described by the OECD and further optimized by the FAO/WHO, is the underlying principle in GM food safety assessment. It was formulated to comparatively analyze the nutrients, anti-nutrients, and other components in GM crops and their non-transgenic comparators (OECD, 2000).

Among the crop nutrients, trace minerals play important roles in human and animal health (Bhullar and Gruissem, 2013). However, trace mineral deficiencies are still common throughout the world. Three main strategies have been proposed to defeat micronutrient malnutrition: food fortification, traditional breeding, and genetic engineering (Lucca et al., 2006). Among them, the transgenic approach is the most efficient at producing micronutrient-fortified crops. Whether genetic modification techniques affect the mineral compositions in the GM crops is an essential part of the biosafety assessment. Depending on whether the transformed genes are directly microelements relevant or not, effects on microelements from GM crops can be divided into two kinds. Thereby, the relationship between particular trace minerals, related exogenous genes and crops' end uses will be discussed, as will the relationship between transgenic technology, main trace minerals, and biological safety.

## Microelements and their genetic regulation via transgenic approach

### Selenium

Interest in selenium (Se) has been growing since it was shown to reduce cancer risk in a landmark trial (Clark et al., 1997). Trace amounts of Se are essential to cellular functions of many organisms, while it is toxic at higher concentrations. Plants show variation in their characteristics of Se uptake, translocation, assimilation, and metabolism, as well as in their tolerance to excessive Se concentrations (White and Broadley, 2005; Gupta and Gupta, 2016). Se accumulators are those plants that can hyperaccumulate Se in their shoots while growing on seleniferous soils, many of which belong to the genus *Astragalus*. Se accumulators are able to accumulate Se from hundreds to several thousand mg kg^−1^ dry weight. However, most crop plants are Se non-accumulators, which accumulate no more than 100 mg Se kg^−1^ dry weight when grown on seleniferous soils and cannot tolerate higher tissue Se concentrations (Brown and Shrift, 1982; White, 2016). The Se uptake ability of agricultural crops largely depends on the plant species; cruciferae usually rank first, then rye grass, leguminosae, and cereals (Bisbjerg and Gissel-Nielsen, 1969). As for wheat, it was found to be more tolerant of Se than tobacco, soybeans or rice. It was exhibited that early root growth of wheat was inhibited when solution concentrations of Se were above 10 mg L^−1^. Interestingly, different forms of Se behave differently. While 70 mg L^−1^ Se concentration was found to inhibit germination for selenite, 150 mg L^−1^ Se concentration failed to repress seed germination for selenate. These results demonstrated the potential of wheat biofortification in Australia as Se phytotoxicity will not be observed in wheat (Lyons et al., 2005; Li H. F. et al., 2008; Wu et al., 2015).

Se shares similar metabolic pathways in plants with sulfur (S) because of their chemical and physical similarities (Shinmachi et al., 2010; Tian et al., 2017). There are two key enzymes involved in Se metabolism pathways: ATP sulfurylase (ATPS) and selenocysteine methyltransferase (SMT). ATPS respectively catalyzes ATP with either sulfate or selenate to form adenosine 5′-phosphosulfate or adenosine 5′-phosphoselenate (Sors et al., 2005; Schiavon et al., 2015). SMT is involved in the synthesis of Se-methylselenocysteine by methylating selenocysteine (SeCys; Neuhierl and Böck, 1996). Simultaneous overexpression of both the ATPS and SMT genes from the Se hyperaccumulator *Astragalus bisulcatus* in Indian mustard resulted in 4–9 times increased accumulation of Se in transgenic lines (LeDuc et al., 2004, 2006). There are many other enzymes involved in Se and S metabolic pathways in addition to APS and SMT. APS reductase is one of them. It participates in the metabolism of selenoamino acids and sulfur by acting on the reduction of APS to sulfite in plants (Suter et al., 2000). Heterologous expression of the APS reductase gene (*PaAPR*) in *Arabidopsis thaliana* resulted in enhanced sulfite reduction, which might indirectly affect Se metabolism (Tsakraklides et al., 2002). Furthermore, selenate reduction has been dramatically increased in transgenic *A. thaliana* by overexpressing both ATPS and APR; thus, the total Se concentration in shoots of transgenic lines was less than in wild type (Sors et al., 2005). However, the relation between APS reductase and APSe reduction remains unclear, and more studies need to be conducted. While these enzymes exhibit various abilities in Se accumulation of plants, which might be applied in Se biofortification, they also play roles in phytoremediation. Overexpression of SMT from *A. bisulcatus* in *A. thaliana* increased selenite tolerance and foliar Se accumulation, and this indicates that it is feasible to allow a Se non-accumulator to accumulate Se-methyl selenocysteine and γ-glutamylmethyl selenocysteine through genetic engineering techniques (Ellis et al., 2004). Therefore, transgenic methods provide a possible approach to generating Se-tolerant plants that could be applied to phytoremediation of Se-contaminated land, and a field trial of transgenic Indian Mustard with the adenosine triphosphate sulfurylase (APS), ç-glutamyl-cysteine synthetase (ECS), and glutathione synthetase (GS) genes overexpressed showed better tolerance to selenium-contaminated sediment (Bañuelos et al., 2005). Although transferring certain genes to plants has obvious effects on the Se accumulation or tolerance of plants, fertilization remains a promising strategy for Se biofortification of crops (White and Broadley, 2009; Wu et al., 2015).

### Boron

Boron (B) is required by both plants and animals. B deficiency has been implicated in human osteoporosis (Nielsen, 1997). As for plants, small amounts of B play a vital role in the cell wall as a component of a B-dimeric rhamnogalacturonan II (RG-II) complex during normal plant growth, and B is also found to be toxic at high concentrations like some other minerals (Miwa and Fujiwara, 2010; Fang et al., 2016). While B deficiency problems can be easily settled by fertilization, high soil B is a major problem. Therefore, new varieties that are tolerant of high B concentrations need to be cultivated.

B uptake is conducted by transport proteins through plant roots. In *Arabidopsis*, there are two main types of transporters localizing in the plasma membrane: BORs and NIPs (nodulin26-like intrinsic proteins). BOR1 efficiently transports B into xylem, and the *A. thaliana* mutant *bor1-1* was found to be more sensitive to B deficiency (Takano et al., 2001, 2002; Uraguchi et al., 2014). Furthermore, transgenic lines overexpressing *AtBOR1* exhibited greater B accumulation in shoots than non-transformants (Wakuta et al., 2016). Unlike BOR1, BOR2 transports B from symplasts to apoplasts, which is necessary for effective cross linking of RG-II in the cell wall, as well as elongation of root cells under B-limited conditions (Miwa et al., 2013). BOR4, a BOR1 homolog, was found in relation to the B concentration in shoots (Miwa et al., 2007; Miwa and Fujiwara, 2011). NIP5:1, which is upregulated under B limitation, differing from BOR family acting in B transport, plays a crucial role in B absorption in plants (Takano et al., 2006), while NIP6:1 facilitates boric acid transfer at the nodal regions of shoots but not roots (Tanaka et al., 2008). These research studies that try to figure out in which way plants are able to tolerate B can be applied to improving crop yields on boron-contaminated soils. Based on the *AtBOR* gene sequences, four rice *BOR-like* genes have been identified, and the activity of GUS as a reporter in transgenic rice varies with cell-type specificity in response to the B content in the medium (Nakagawa et al., 2007). Likewise, genes expressing borate transporters in wheat as well as barley have been reported (Reid, 2007). Furthermore, overexpression of *HvBor1a* encoding a barley transporter in tobacco suggested that the *HvBor1a* gene might be a promising gene for enhancing the B tolerance of crop plants (Gümüşel et al., 2012). In addition to BORs and NIPs, members of the PIP subfamily also participate in B transport. PIPs are plasma membrane intrinsic proteins. Maize aquaporins *ZmPIP1* and *ZmPIP2* were shown to be involved in transporting B (Martinez-Ballesta et al., 2008). Two *PIP* genes in rice, namely *OsPIP2;4* and *OsPIP2;7*, were characterized as being involved in B permeability and tolerance, overexpression of which in Arabidopsis resulted in higher tolerance under B toxicity (Kumar et al., 2014). Similar results appeared from other aquaporins members, Arabidopsis heterologously expressing *OsPIP1;3* and *OsPIP2;6* showed increased tolerance to B toxicity through both B influx and efflux transport (Mosa et al., 2016), making it useful to manipulate PIPs in crops in improving B tolerance in crops. In addition, transgenic rice with enhanced sorbitol synthesis was found to facilitate the remobilization of B in phloem (Bellaloui et al., 2010). However, it still remains a great concern to increase yield by engineering tolerance to B toxicity in crops by the transgenic approach (Reid, 2010).

### Iron

Iron is an essential microelement for many organisms including plant species, particularly for humans. Iron deficiency anemia is considered the most common type of anemia, and it can impact work efficiency, impair thermoregulation, and cause immune dysfunction in adults (Clark, 2008). Iron deficiency can induce the inhibition of plant growth as well (Bocchini et al., 2015). Iron, when its excess is not great, presents predominantly in the form of a water-soluble iron protein called ferritin (Zielinska-Dawidziak, 2015). Ferritin is an iron-storage protein existing widely in animals, plants and bacteria that consists of 24 protein subunits and can store ~4,000 iron atoms as a hollow globular protein complex (Vigani et al., 2013). Ferritin is considered to play at least two major parts in plants: firstly, as a source of iron required by metal enzymes involved in photosynthesis and other respiratory processes, and secondly, as a defense component, protecting cells from the toxic effects of overloaded iron (Briat et al., 2010). After transferring soybean ferritin cDNA to tobacco via *Agrobacterium tumefaciens*, iron accumulation of transformant leaves was up to 30% higher than that of wild type, and these results provided evidence of breeding high iron content crops by transferring the ferritin gene (Goto et al., 1998). By introducing the soybean ferritin gene into rice, high iron content transgenic lines were generated. The iron content of self-pollinated T_1_ seeds was as much as triple that of the control line (Goto et al., 1999). Further study showed that transgenic wheat and rice constitutively expressing the soybean ferritin gene via particle bombardment had increased iron content in vegetative tissues instead of seeds, suggesting that in order to accomplish ferritin overexpression and iron accumulation in transgenic seeds, seed-specific promoters such as Gt-1 and Glu-B1 should be applied (Drakakaki et al., 2000). Through overexpression of the soybean ferritin *SoyferH-1* gene with a strong endosperm-specific promoter, the storage ability of iron in rice seeds has been improved; nevertheless, an increase in Fe content failed to parallel the higher expression level of the foreign ferritin gene (Qu et al., 2005). Interestingly, increased expression of the endogenous *NtFer1* gene was greatly correlated with that of an exogenous *TaFer1* gene in transgenic tobacco, suggesting interactions between different ferritin subunits (Wang J. et al., 2015; Yao et al., 2016).

Phytic acid is among the main inhibitory factors of iron availability; in other words, iron absorption may be extremely low due to inhibitors (Hurrell et al., 2003). Transgenic wheat and rice that constitutively express heterogenous phytase have been generated (Brinch-Pedersen et al., 2000, 2003; Hong et al., 2004). *Aspergillus* phytase and soybean ferritin have been transferred to maize separately or together, with the hope of increasing the bioavailable iron content in the endosperm. The result demonstrated that the heterologous expression of ferritin and phytase in crops could contribute to an increase in iron bioavailability and absorption, especially in cereal-based diets that more or less lack some trace elements (Drakakaki et al., 2005). It is worth noting that proteomic analysis of phytase transgenic corn seeds showed that some ribosomal proteins and heat-shock proteins might present adaptive effects in response to the insertion of *Aspergillus* phytase *phyA2* (Tan et al., 2017). Three ways have been proposed to increase the accumulation of iron in rice grains. The first is to insert a *P. vulgaris* ferritin gene into rice grains to enhance iron accumulation and an *A. fumigatus* heat-tolerant phytase into the rice endosperm to improve its bioavailability. Second, an endogenous gene encoding a cysteine-rich metallothionein-like protein was overexpressed to increase the absorption of iron. Finally, these different transgenic lines were hybridized to introduce new quality improvements, further substantially improving iron nutrition in daily diets of rice (Lucca et al., 2001).

### Zinc

As an essential trace element for plants, humans, and microorganisms, zinc (Zn) participates in various biochemical pathways. Zn deficiency is one of the most severe problems threatening as much as one-quarter of the world's population. Nevertheless, excess Zn can also be toxic (Broadley et al., 2007). The ZIP family, as one of the Zn transporter families, can transport not only Zn but a variety of cations, including Ca, Fe, and Mg, and it is considered to be part of the primary uptake of Zn in plants (Guerinot, 2000). Several transporters of this family in rice have been characterized, such as OsZIP4, OsZIP5, and OsZIP8. Overexpression of the *OsZIP4* gene in rice resulted in the 10-fold accumulation of Zn concentration compared with vector controls in roots, while it was five times lower in shoots and four times lower in seeds, indicating that overexpression of *OsZIP4* disrupts Zn distribution in rice (Ishimaru et al., 2007). Overexpression of the *OsZIP5* gene caused a decrease in shoot Zn concentration but an increase in the roots, indicating that OsZIP5 transporter might be involved in root to shoot translocation (Lee et al., 2010a). OsZIP8 is also a plasma membrane zinc transporter that participates in Zn uptake and translocation. Overexpression of the *OsZIP8* gene resulted in a decrease in Zn levels in shoots as well as mature seeds, but the opposite was found in roots (Lee et al., 2010b). In addition, transgenic barley overexpressing *Arabidopsis* zinc transporter *AtZIP1* revealed increases in both zinc and iron contents in seeds (Ramesh et al., 2004). *ZmZIP3* was introduced into Arabidopsis, leading to increased Zn concentration in roots but a decrease in shoots (Li et al., 2015). Ferritin protein is also found to take part in the accumulation of Zn in addition to Fe (Vasconcelos et al., 2003; Paul et al., 2012; Liu et al., 2016). Heterologous expression of the soybean ferritin gene in transgenic rice grains results in high iron and zinc levels (Vasconcelos et al., 2003). Metal transporters might be of great value in increasing zinc and iron contents, to further improve the micronutrient content of crops. However, while the Fe and Zn concentrations increased in wheat grains heterologously expressing sickle alfalfa *ferritin*, the grain Cu and Cd concentrations decreased significantly (Liu et al., 2016). Additionally, phytosiderophores (MAs) and nicotianamine (NA), which are members of the mugineic acid family, also have important roles in the uptake and distribution of Zn and iron in plants (Ricachenevsky et al., 2013; Wang et al., 2013). Zn and iron concentrations have been decreased *via* expressing barley genes involved in the biosynthesis of Mas (Takahashi et al., 2003), and transgenic rice with the barley NA synthase gene *HvNAS1* displayed increased endogenous NA and MA contents and subsequently increased iron and zinc concentrations in grain (Masuda et al., 2009).

### Copper

Copper (Cu), another micronutrient necessary to plants and humans, is nonetheless toxic at elevated levels. The copper transporter (CTR) family is one type of high-affinity Cu transport protein that contains three predicted transmembrane segments (Burkhead et al., 2009). The COPT proteins are transporters of the CTR family, and five members (COPT1–5) have been identified in *A. thaliana*. In plants, mRNA levels of COPT1 and COPT2 were strongly reduced, while those of COPT3, COPT4, and COPT5 were apparently unaffected in response to Cu treatment of leaves (Sancenón et al., 2003). COPT1 antisense transgenic *Arabidopsis* showed reduced ^64^Cu transport rates in seedlings compared with the controls (Sancenón et al., 2004). Transgenic tobacco hairy roots expressing a bacterial *copC* gene could remove copper from aqueous solutions by rhizofiltration (Pérez-Palacios et al., 2017). Members of the heavy metal P_1B_-ATPase (HMA) superfamily function to remove Cu from the cytoplasm. AtHMA1, which belongs to the HMA family, affects Cu homeostasis in yeast expression experiments. *Arabidopsis hma1* mutants exhibited reduced Cu content in cell chloroplasts and presented a photosensitivity phenotype under high light compared with the wild type (Seigneurin-Berny et al., 2006). *OsHMA9* knockout rice exhibited increased Cu, Zn, Pb, and Cd uptake, while they were much more sensitive to excessive Cu, Zn, and Pb (Lee et al., 2007). In addition, plants and other organisms can also remove elevated Cu within the cell through a type of metal-binding protein called metallochaperones (MTs). Transgenic tobacco with the yeast metallothionein gene *CUP1* exhibited increased copper uptake from contaminated soil (Thomas et al., 2003). Metallothioneins are also involved in ROS scavenging; Arabidopsis overexpressing *OsMT2c* exhibited enhanced tolerance to Cu stress due to increased ROS scavenging compared with the controls (Liu et al., 2015).

Particular minerals and transformation of certain genes have been discussed above, and these have been summarized and described in Table 1.

**Table 1:** Summary of minerals and transformation of certain genes.

**Minerals**	**Plants species**	**Genes**	**Performances**	**References**
Se	Arabidopsis and Indian mustard	*SMT* gene from *A. bisulcatus*	Increased Se tolerance and accumulation	LeDuc et al., 2004
	Arabidopsis	*SMT* from *A. bisulcatus*	Accumulated MeSeCys and γ-GluMeSeCys in shoots	Ellis et al., 2004
	Arabidopsis	*ATPS* from *P. aeruginosa* and *APR* from *A. thaliana*	Produced an additive effect on selenate reduction	Sors et al., 2005
	Indian mustard	*APS* and *SMT* from *A. bisulcatus*	Greater uptake and conversion of selenite into MetSeCys	LeDuc et al., 2006
B	Arabidopsis	*AtBOR1*	Increased B concentration in the shoots	Wakuta et al., 2016
	Arabidopsis	*OsPIP2;4* and *OsPIP2;7*	Imparted higher tolerance under B toxicity	Kumar et al., 2014
	Arabidopsis	*OsPIP1;3 and OsPIP2;6*	Enhanced tolerance to B toxicity	Mosa et al., 2016
	Tobacco	*HvBOR1a*	Might be of great value in engineering tolerance to B toxicity	Gümüşel et al., 2012
	Rice	*S6PDH* from apple	Remobilization of B from mature leaves to flag leaves	Bellaloui et al., 2010
Fe	Tobacco	Soybean ferritin with CaMV 35S promoter	Increased iron content	Goto et al., 1998
	Tobacco	A ferritin gene from *T. androssowii, TaFer1*	Showed a higher tolerance under low-iron conditions	Yao et al., 2016
	Rice	Soybean ferritin with rice GluB-1 promoter	3-fold iron content	Goto et al., 1999
	Rice	Genes from *S. ruminantium* (*SrPf6*) and *E. coli* (*appA*)	Presented 46–60 times the phytase activity	Hong et al., 2004
	Rice	Soybean ferritin *SoyferH-1* gene	Decreased mean Fe concentration in leaves	Qu et al., 2005
	Maize	Soybean ferritin and *Aspergillus* phytase genes	Significant increase in bioavailable iron	Drakakaki et al., 2005
	Maize	*Aspergillus* phytase genes *phyA2*	Ribosomal proteins and heat-shock proteins generated adaptive effects	Tan et al., 2017
	Wheat and Rice	Soybean ferritin with constitutive maize ubiquitin-1 promoter	Significantly increases iron levels in vegetative tissues	Drakakaki et al., 2000
	Wheat	*Aspergillus niger* phytase-encoding gene (phyA) with maize *ubiquitin*-1 promoter	Up to a 4-fold increase in phytase activity	Brinch-Pedersen et al., 2000
	Wheat	*Aspergillus* phytase with wheat 1Dx5 promoter	Efficient degradation of InsP6	Brinch-Pedersen et al., 2003
Zn	Arabidopsis	*ZmIRT1* or *ZmZIP3*	Showed altered tolerance to various Fe and Zn conditions	Li et al., 2015
	Tobacco	*Hvnaat*	Lower concentrations of all four metals in young leaves	Takahashi et al., 2003
	Rice	soybean *ferritin* gene with glutelin promoter	Enhanced iron and zinc accumulation	Vasconcelos et al., 2003
	Rice	*OsZIP4*	10 times higher Zn concentration in root, but five times lower in shoot	Ishimaru et al., 2007
	Rice	*HvNAS1*	Increased iron and zinc concentrations in grains	Masuda et al., 2009
	Rice	*OsZIP5*	Decreased Zn concentration in shoots, but increased in the roots	Lee et al., 2010a
	Rice	*OsZIP8*	Lower levels in shoots and mature seeds, but an increase in the roots	Lee et al., 2010b
	Rice	*Osfer2*	2.09- and 1.37-fold iron and zinc accumulation, respectively	Paul et al., 2012
	Barley	*AtZIP1*	Increases short-term zinc uptake after zinc deprivation and seed zinc content	Ramesh et al., 2004
	Wheat	Sickle alfalfa *ferritin*	Increases the grain Fe and Zn concentrations	Liu et al., 2016
Cu	Arabidopsis	*AtHMA1*	Did not display any obvious phenotype under standard conditions	Seigneurin-Berny et al., 2006
	Arabidopsis	*OsMT2c*	Improved tolerance to Cu stress	Liu et al., 2015
	Tobacco	Yeast metallothionein gene *CUP1*	Promotes copper uptake from contaminated soils	Thomas et al., 2003
	Tobacco	*copC* from *Pseudomonas fluorescens*	Accumulated twice the amount of copper	Pérez-Palacios et al., 2017
	Rice	*OsHMA9*	Accumulated more Cu, Zn, Pb, and Cd in the shoots	Lee et al., 2007

## Transgenes and their impact on minerals

According to the 2016 report of the ISAAA, the global acreage of GM crops has increased 100-fold from the previous 1.7 million hectares in 1996 to 185.1 million hectares in 2016, indicating that biotech crops are the fastest growing and fastest adopted crop technology in recent years (http://www.isaaa.org/inbrief/default.asp). Meanwhile, lots of fundamental research is being conducted all over the world. As for wheat, many genes involved in good processing and nutritional quality have been transferred into wheat on a large scale, such as high-molecular-weight glutenin subunit (HMW-GS) genes *1Ax1, avenin-like b* gene (Wang et al., 2010; Li et al., 2012; Ma et al., 2013; Ellis et al., 2014; Jin et al., 2015; Han et al., 2017). However, whether gene transformation and expression will cause unintended compositional changes while producing improved end-products in these GM wheat varieties remains unclear. These unintended compositional changes may include mineral changes and secondary metabolite changes. Take transgenic wheat with *1Ax1* overexpression for example (Wang et al., 2010), the excess accumulation of HMW-GS formed by intermolecular disulfide bond, might indirectly affect the selenium homeostasis due to the sophisticated interactions of molecules in cells of transgenic wheat, which was implied in *Stanleya pinnata* (El Mehdawi et al., 2018). It is worth mentioning that hybridization breeding efforts have also vastly enhanced HMW-GS content of wheat, yet whether one variable of single gene insertion or multivariate of vast genes on chromosomes has more effect on minerals content or functions is unknown. Therefore, food made from transgenic crops must undergo standard safety assessment to guarantee that no allergenicity or toxicity to humans or animals exists.

Many disease-resistant crops have been generated, with the *OsCK1* GM rice among them. A choline kinase (*CK1*) gene isolated from *Oryza sativa* and the phosphinothricin acetyltransferase (*PAT*) gene from *Streptomyces hygroscopicus* have simultaneously been expressed in the *OsCK1* GM rice (Lee et al., 2005). A comparative analysis of *OsCK1* GM rice and its corresponding controls together with two commercial rice varieties was performed in two different locations in Korea (Park et al., 2015; Oh et al., 2016). In these results, significant differences between GM rice and its conventional counterpart were found for three minerals (Mg, P, and Na; Park et al., 2015). However, these did not go beyond the ranges of values formulated by the OECD. Another study that analyzed levels of phytic acid, which participates in mineral absorption, revealed that GM rice varied significantly from non-GM rice at the phytic acid level. However, these values in GM rice were still within the respective tolerance interval (TI) range (Oh et al., 2015). The application of TIs in statistical analysis was to assess the effects caused by environmental variants. Therefore, the mineral contents were impacted by the environmental factors rather than genetic modification. In view of substantial equivalent analysis, the mineral components of the GM rice should be equivalent to those of non-GM rice varieties (Oh et al., 2016). A similar analysis was performed to assess the safety of a drought-tolerant GM rice harboring the *CaMsrB2* gene (Cho et al., 2016). The sodium content in the GM rice was 1.24-fold of that in the non-GM controls. Nevertheless, the statistical analysis proved that the difference was more likely caused by the regional environment (Gayen et al., 2016). Moreover, analysis of a transgenic rice variety overexpressing the disease resistance gene *Xa21* showed that the contents of the main minerals in the GM rice were equal to those in the conventional rice controls (Gayen et al., 2016). The same assessment results were also found in other transgenic rice with exogenous genes (Li et al., 2007; Li X. et al., 2008; Park et al., 2012).

As substantial equivalence is the concept that is most widely adopted and used to evaluate the biosafety of GM crops, compositional safety has been investigated by comparing transgenic crops like soybean, pepper, cotton, and corn with non-transgenic counterparts (Ridley et al., 2002; Park et al., 2006; McCann et al., 2007; Rui et al., 2009; Herman et al., 2010, 2011, 2013; Mohanta et al., 2011; Costa et al., 2015). Most of these studies draw a similar conclusion that there were no statistical differences between GM plants and their controls. Even though the concentrations of exceptional microelements in GM cotton (Herman et al., 2013), rice (Li X. et al., 2008; Oh et al., 2015), and corn (Herman et al., 2010) changed remarkably, they were still within the respective 99% TIs. Nevertheless, the vast majority of these studies only focused on the composition of minerals when making comparative analysis of GM crops while paying little attention to the distribution changes in transgenic plants, because different tissues or organs of the same plant species differ in mineral absorption and accumulation. A study on the distribution pattern of 12 minerals including heavy metals in GM cotton (*Bt*+*CpTI*) revealed that the transgenic cotton accumulated less Cd and As than common cotton in all organs but lower Pb in the stem (Rui and Qu, 2009).

Nonetheless, there are many other concerns, such as mineral uptake and allocation acting reciprocally. One ABC transporter in *S. pyogenes* has affinity for three metal minerals: Zn, Fe, and Cu. Moreover, Zn and Cu were detected to competitively bind to the same site (Janulczyk et al., 1999). In previous studies, the Fe/Zn accumulation in rice endosperm was fortified by overexpressing genes participating in Fe uptake, translocation, and storage (Kobayashi and Nishizawa, 2012; Lee et al., 2012). Heteroexpression of a soybean ferritin gene gave rise to iron storage ability in rice seeds, while no obvious differences in other divalent-metal concentrations were displayed in the seeds comparing all transgenic lines and wild types (Qu et al., 2005). Safety concerns have arisen in molecular Fe/Zn fortification because the cadmium can be concurrently taken up through the Fe uptake system (Nakanishi et al., 2006). *MxIRT1* has been introduced into rice to alleviate the Fe deficiency, and this transformation unexpectedly induced Cd uptake on trial. But it is worth noting that MxIRT1 takes up the lowest Cd in comparison with AtIRT1 and OsIRT1 in transgenic protoplasts (Tan et al., 2015). Another investigation on genetic Zn fortification grains reported that the Cd content was slightly higher in GM crops than in control crops, however this result was still under the toxic threshold (Zhang et al., 2012). Since the Cd uptake pathway in plants has been identified before (Uraguchi et al., 2009, 2011), genetic modification can be used to prevent excess Cd accumulation by depressing expression of genes functioning in Cd uptake (Ishikawa et al., 2012; Ishimaru et al., 2012).

In addition, the effects of genetic modification and growth environment have been evaluated simultaneously. While 20 to 22 protein levels differentially changed in transgenic rice seeds comparing to wild types, 21 proteins levels were found to be higher or lower as a consequence of growth environment. These results suggested that the impact of the single gene insertion into the genome on the nutrient quality of crops was no more than that of the growth environment (Wang et al., 2012). Another study also revealed that bacterial inoculation enhanced selenium and iron concentration in wheat (Yasin et al., 2015). As traditional fertilization is a simple and easy way to handle soil microelement deficiencies, effect of basic fertilizer on transgenic cereals has been assessed as well. A significant reduction in AMF (arbuscular mycorrhizal fungal) colonization in *Bt11* maize roots emerged with limited fertilizer, while no evident difference was found between the *Bt11* and the control with appropriate fertilizer (Cheeke et al., 2011). Similar research was seldom reported in mineral related transgenic crops, which might be due to the rare licensed and mineral relevant GM crops.

All the transgenic crops mentioned above have been listed and described in Table 2.

**Table 2:** Transgenes and their impact on minerals.

**GM plants**	**Performance**	**Genes**	**Impact on minerals**	**References**
Pepper	Herbicide-tolerant	phosphinothricin acetyltransferase gene	No significant difference	Park et al., 2006
Soybean	Herbicide-tolerant	AAD-12 enzyme gene from *D. acidovorans*	Only calcium levels in the transformant significantly differed due to the effect of the weather stress	Herman et al., 2011
Soybean	Roundup ready	Roundup Ready gene	No statistical difference	Costa et al., 2015
Cotton	Herbicide-tolerant	*aad-12* and *pat* genes	The manganese level was 15% lower in the transgenic entry compared with the isoline	Herman et al., 2013
Cotton	Insect-resistant	*Bt* and cowpea trypsin inhibitor *(CpTI)*	Absorption and distribution of 12 mineral elements, especially K, P, Fe, and Si, changed significantly	Rui et al., 2009
Cotton	Insect-resistant	*Bt* and cowpea trypsin inhibitor *(CpTI)*	Insertion of foreign gene (Bt) might change the absorbing dynamics of most heavy metals.	Rui et al., 2009
Cotton	Insect-resistant	*cry1C* gene from *B. thuringiensis*	Mineral composition and heavy mineral content were similar	Mohanta et al., 2011
Rice	higher iron and zinc concentration in seeds	Apple *MxIRT1*	Decreased influx ability under optimum Fe conditions	Janulczyk et al., 1999
Rice	ferritin hyper-expressing	Soybean ferritin gene	No obvious differences were observed.	Qu et al., 2005
Rice	Herbicide-tolerant	*bar* gene from *S. hygroscopicus*	Statistically significant differences were observed for iron between *Bar68-1* rice and non-transgenic rice	Li et al., 2008
Rice	Insect-resistant	Cowpea trypsin inhibitor gene *sck* and *cry1Ac* from *B. thuringiensis*	All significantly different mean values for IRR rice were within commercial rice reference ranges	Li et al., 2007
Rice	Insect-resistant	*cry1Ac* gene isolated from *B. thuringiensis*	The values were in good compliance with reference ranges provided by the OECD	Park et al., 2012
Rice	Disease-resistant	*OsCK1* and *PAT* genes from *S. hygroscopicus*	With the exception of zinc, sulfur, and phosphorus, none of the minerals differed significantly	Park et al., 2015
Rice	Disease-resistant	*OsCK1* and *PAT* genes from *S. hygroscopicus*	The values for these components in GM rice were within the respective 99% Tis	Oh et al., 2015
Rice	Drought-resistant	The pepper methionine sulfoxide reductase B2 gene *CaMsrB2*	No differences with respect to the whole nutritional composition	Cho et al., 2016
Rice	Bacterial blight-resistant	*Xa21* gene	Only small variations in the Na, Fe, and Zn compositions within each seed type	Gayen et al., 2016
Maize	Glyphosate-tolerant	*cp4 epsps* gene from *Agrobacterium*	Minerals in the grain were comparable to those in non-transgenic control	Ridley et al., 2002
Maize	Insect-protected and glyphosate-tolerant	*cp4 epsps* gene from *Agrobacterium* and *cry3Bb1* from *B. thuringiensis*	No statistical differences	McCann et al., 2007
Maize	Herbicide-tolerant	Aryloxyalkanoate dioxygenase-1 enzyme gene from *S. herbicidovorans*	Calcium levels for the transgenic entries were statistically higher	Herman et al., 2010

## Discussion and future prospects

Transgenic technology has brought many promising GM plants with improved quality and/or enhanced stress tolerance and has also allowed microelement non-accumulators to accumulate trace minerals and common plants to hyperaccumulate them. Lots of work has been done to compare GM crops and their non-transgenic counterparts (Yang et al., 2013; Wang L. et al., 2015). These comparative analyses were mostly conducted by proteomics methods, which mainly use two-dimensional electrophoresis (2-DE) coupled with mass spectrometry (MS) to distinguish differential proteins or protein content between GM crops and their wild types (Barros et al., 2010; Brandão et al., 2010; Yang et al., 2013; Wang L. et al., 2015). Metabolomics techniques such as HPLC, NMR, and GC/MS have also been used to analyze differences between metabolites including carbohydrates, lipids, and amino acids (Brandão et al., 2010). In addition, other compositions containing amino acids, fatty acids, minerals, vitamins and anti-nutritive components have been analyzed to investigate the biosafety of GM crops (Li et al., 2007; Li X. et al., 2008; Barros et al., 2010; Wang et al., 2012; Gayen et al., 2013).

However, there are more details that should be considered and improved in these studies. When transforming microelement transport- or metabolism-related genes, not only the particular one but other minerals have to be analyzed. Even the distribution pattern of the microelement including the composition changes should be considered. Besides, microelements exist in various forms in plants, the bioavailable forms and biologically active precursors need to be analyzed separately. Apart from the analysis of samples, the treatment of plants could be diverse and correlated in parallel with the performance of GM plants, for instance, comparative analysis between drought-resistant GM rice and conventional rice should be taken from both common circumstances and drought stress treatment. Certainly, all of the GM plants should be demonstrated in field trials, but still, stress treatment should be taken into consideration during safety assessment. In addition to nutritional quality, growth environment and fertilization way should be taken into consideration in later studies on GM cereals. Therefore, various approaches including genetic engineering, fertilization, hybridization should be integrated and complemented each other to accomplish biofortification of plants.

With the aid of transgenic techniques, it is feasible to manipulate cereal plants to improve or tolerate concentrated minerals. However, we should guarantee that the transgenic cereals are safe in both environmental friendliness and end-use properties, especially in mineral metabolism respects. Based on the requested motivations of biosafety issues, systematic design and analysis of transgenic cereal should be investigated comprehensively at different levels of gene confirmation, genomics, proteomics, metabolomics, nutritiomics, and the absorption, metabolism, and function of minerals.

## Author contributions

XY, QL, KH collected materials; XY wrote the draft manuscript; GY and GH revised the manuscript.

### Conflict of interest statement

The authors declare that the research was conducted in the absence of any commercial or financial relationships that could be construed as a potential conflict of interest.
